# Predictors and barriers to minimally adequate treatment among treated individuals with mental disorders: results from the World Mental Health Surveys

**DOI:** 10.1186/s13033-025-00686-6

**Published:** 2025-12-18

**Authors:** Julia R. Pozuelo, Daniel V. Vigo, Alan E. Kazdin, Meredith G. Harris, Dan J. Stein, Maria Carmen Viana, Irving Hwang, Timothy L. Kessler, Sophie M. Manoukian, Nancy A. Sampson, Sergio Aguilar-Gaxiola, Jordi Alonso, Laura Helena Andrade, Olatunde O. Ayinde, Ronny Bruffaerts, Brendan Bunting, José Miguel Caldas-de-Almeida, Stephanie Chardoul, Giovanni de Girolamo, Cristina Domenech, Oye Gureje, Elie G. Karam, Andrzej Kiejna, Viviane Kovess-Masfety, Maria Elena Medina-Mora, Jacek Moskalewicz, Fernando Navarro-Mateu, Daisuke Nishi, Marina Piazza, José Posada-Villa, Kate M. Scott, Margreet ten Have, Yolanda Torres, Cristian Vladescu, Ronald C. Kessler, World Mental Health Survey collaborators, Ali Al-Hamzawi, Yasmin A. Altwaijri, Lukoye Atwoli, Corina Benjet, Guilherme Borges, Evelyn J. Bromet, Jose Miguel Caldas-de-Almeida, Graça Cardoso, Alfredo H. Cía, Louisa Degenhardt, Josep Maria Haro, Hristo Hinkov, Chi-yi Hu, Peter de Jonge, Aimee Nasser Karam, Georges Karam, Norito Kawakami, John J. McGrath, Annelieke Roest, Juan Carlos Stagnaro, Nathan Tintle, Cristian Vladescu, David R. Williams, Bogdan Wojtyniak, Miguel Xavier, Alan M. Zaslavsky

**Affiliations:** 1 Department of Global Health and Social Medicine, Harvard Medical School, 641 Huntington Ave, Boston, 02115 MA USA; 2 Department of Psychiatry, University of Oxford, Oxford, UK; 3 MRC/Wits Rural Public Health and Health Transitions Research Unit (Agincourt), School of Public Health, Faculty of Health Sciences, University of the Witwatersrand, Johannesburg, South Africa; 4 Department of Psychiatry & School of Population and Public Health, Faculty of Medicine, University of British Columbia, UBC Hospital – Detwiller Pavilion, UBC Vancouver Campus, Room 2813, 2255 Wesbrook Mall, Vancouver, V6T 2A1 BC Canada; 5 Department of Psychology, Yale University, 2 Hillhouse Avenue- 208205, New Haven, 06520 CT USA; 6 School of Public Health, The University of Queensland, C/O QCMHR, Locked Bag 500, Archerfield, 4108 QLD Australia; 7 Queensland Centre for Mental Health Research, The Park Centre for Mental Health, Wolston Park Rd, Wacol , Brisbane, 4076 QLD Australia; 8 Department of Psychiatry & Mental Health and South African Medical Council Research Unit On Risk and Resilience in Mental Disorders, University of Cape Town, Rondebosch, Cape Town, 7925 ZA South Africa; 9 Department of Social Medicine, Postgraduate Program in Public Health, Federal University of Espírito Santo, Av. Marechal Campos, 1468CEP 29.043-900, Vitória, Espírito Santo (ES) Brazil; 10 Department of Health Care Policy, Harvard Medical School, 180 Longwood Avenue, Boston, 02115 MA USA; 11 The Watson Institute for International and Public Affairs, Brown University, 111 Thayer St., Providence, 02912 RI USA; 12 Department of Internal Medicine, Center for Reducing Health Disparities, UC Davis Health System, 2921 Stockton Blvd., Suite 1408, Sacramento, 95817 CA USA; 13 IMIM-Hospital del Mar Medical Research Institute, PRBB Building, Doctor Aiguader, 88, Barcelona, 08003 Spain; 14 CIBER en Epidemiología y Salud Pública (CIBERESP), Av. Monforte de Lemos, 3-5, Pabellón 11, Planta 0, Madrid, 28029 Spain; 15 Pompeu Fabra University (UPF), Plaça de La Mercè, 10-12, Barcelona, 08002 Spain; 16 University of São Paulo Medical School, Núcleo de Epidemiologia Psiquiátrica - LIM 23, Rua Dr., Ovidio Pires de Campos, São Paulo, 78505403-010 CEP Brazil; 17 Department of Psychiatry, University of Ibadan, University College Hospital, Ibadan, 5116 PMB Nigeria; 18 Universitair Psychiatrisch Centrum, Katholieke Universiteit Leuven (KUL), Campus Gasthuisberg, Herestraat 49, B-3000 Louvain, Belgium; 19 School of Psychology, Ulster University, Coleraine Campus, Coleraine, BT52 1SA UK; 20 Lisbon Institute for Global Mental Health, Comprehensive Health Research Centre Nova University of Lisbon, Rua Do Instituto Bacteriológico, 5, Lisbon, 1150-190 Portugal; 21 Survey Research Center, Institute for Social Research, University of Michigan, 330 Packard, Room G358, Ann Arbor, 48104 MI USA; 22 IRCCS Istituto Centro San Giovanni Di Dio Fatebenefratelli, Via Pilastroni 4, Brescia, Italy; 23 Parc Sanitari Sant Joan de Déu, Dr. Antoni Pujadas, 42, Sant Boi de Llobregat, 08830 Barcelona Spain; 24 Department of Psychiatry, Stellenbosch University, Stellenbosch, South Africa; 25 Institute for Development Research Advocacy and Applied Care (IDRAAC), Achrafieh, St. George Hospital Street, Beirut, Lebanon; 26 Department of Psychiatry and Clinical Psychology, St George Hospital University Medical Center, Ashrafieh, Beirut, 166378 Lebanon; 27 Faculty of Applied Studies, University of Lower Silesia, Ul. Strzegomska 55, Wrocław, 53-611 Poland; 28 Institut de Psychologie, UR 4057, Université Paris Cité, 71 avenue Édouard Vaillant, Boulogne Billancourt, 92100 Paris France; 29 National Institute of Psychiatry Ramón de La Fuente Muñiz, Calz. Mexico-Xochimilco 101, San Lorenzo Huipulco, 14370 Ciudad de Mexico, Mexico; 30 Seminar of Studies On Globality, National Autonomous University of Mexico (UNAM), Mexico City, Mexico; 31 Faculty of Psychology, National Autonomous University of Mexico (UNAM), Mexico City, Mexico; 32 Institute of Psychiatry and Neurology, Sobieskiego 9, Warsaw, 02-957 Poland; 33 Unidad de Docencia, Investigacion y Formación en Salud Mental (UDIF-SM), Servicio Murciano de Salud, Murcia Health Service, C/Lorca, nº 58. -El Palmar, Murcia, 30120 Spain; 34 Instituto Murciano de Investigación Biosanitaria - Pascual Parrilla (IMIB-Pascual Parrilla), Hospital Virgen de La Arrixaca, El Palmar, 30120 Murcia Spain; 35 Centro de Investigación Biomédica en ERed en Epidemíologia y Salud Pública (CIBERESP), Madrid, 28029 Spain; 36 Department of Mental Health, Graduate School of Medicine, The University of Tokyo, 7-3-1, Hongo, Bunkyo-Ku, Tokyo, 113-0033 Japan; 37 Facultad de Salud Pública y Administración, Universidad Peruana Cayetano Heredia, Av. Honorio Delgado 430, San Martín de Porres, 15102 Lima, Peru; 38 Colombian Institute of the Nervous System, Clinica Montserrat University Hospital, Calle 134 No. 17-71, Bogotá, Colombia; 39 Department of Psychological Medicine, University of Otago, PO Box 56, Dunedin, 9054 New Zealand; 40 Trimbos-Instituut, Netherlands Institute of Mental Health and Addiction, Da Costakade 45 Postbus 725, Utrecht, 3500, 3521 VS The Netherlands; 41 Center for Excellence On Research in Mental Health, CES University, Medellín, Colombia; 42 National Institute for Health Services Management, 31 Vaselor Str., Bucharest, 21253 Romania; 43 University Titu Maiorescu, 67 A Gheorghe Petraşcu Str., Bucharest, 031593 Romania

**Keywords:** Adequacy of treatment, Cross-national, Mental disorders treatment, Minimally adequate treatment, World mental health survey consortium

## Abstract

**Background:**

Treatments for mental disorders vary widely in type and quality, with many patients failing to receive treatments that meet even minimally adequate standards. We use data from the World Mental Health (WMH) surveys to investigate this variation by examining the prevalence and correlates of minimally adequate treatment (MAT) among patients receiving treatment for common mental disorders.

**Methods:**

Data comes from 25 WMH cross-sectional surveys implemented in 21 countries (n = 1,838 respondents with n = 3,538 12-month treated disorders). MAT was defined according to widely used criteria: pharmacotherapy (≥ 1 month of medication with ≥ four visits to a healthcare provider) or counseling (≥ eight sessions with any provider). Multivariable regression analyses were used to examine associations of socio-demographic, disorder-related, and treatment-related factors with MAT.

**Results:**

Approximately two-thirds (66.2%) of treated cases met MAT criteria. There was limited variation in MAT prevalence across disorder types, number of disorders, or years since disorder onset, but MAT prevalence was positively associated with increased disorder severity. Socio-demographic differences were nonsignificant. Relatively substantial differences in MAT prevalence were found by treatment sector (highest MAT prevalence among patients treated by mental health specialists and those treated by multiple provider types). Further analysis showed that these associations were explained by differences in premature discontinuation, completion of a full course of treatment that did not qualify as MAT, and still being in treatment at the time of interview that did not yet qualify as MAT. Low perceived disorder severity unrelated to more objective measures of severity was a central factor in accounting for premature discontinuation.

**Conclusions:**

While approximately two-thirds of treated cases meet MAT criteria, significant gaps remain involving both premature discontinuation and cases where respondents reported completing a ‘full recommended course of treatment’ that did not involve enough visits or medication duration to meet the MAT standards. Expanding access to mental health specialty providers and increasing patient education about disorder severity would be useful in increasing the proportion of treated cases that receive MAT. Future research should focus on validating MAT definitions against clinical outcomes, standardizing assessment frameworks, and exploring provider- and system-level determinants of treatment adequacy.

**Supplementary Information:**

The online version contains supplementary material available at 10.1186/s13033-025-00686-6.

## Background

Mental disorders are prevalent worldwide, affecting 20–25% of individuals in most countries in a year and up to 50% over their lifetime [1]. The high prevalence, chronicity, and comorbidity of these disorders with other health problems impose significant economic and social burdens both on individuals with these disorders and on society and increase risk of early mortality [2–5]. Despite the growing availability of cost-effective treatments [6–9], access to care for mental disorders remains limited [10–12]. Barriers such as stigma and financial constraints are major obstacles to accessing care [12–18]. Moreover, many patients who obtain care discontinue prematurely [19–22], while those who complete treatment often receive care that falls short of adequate standards [15, 23–27]. This combination of barriers, early dropout, and inadequate care limits the proportion of people with mental disorders who receive effective treatment [10].

The concept of Minimally Adequate Treatment (MAT) was developed as a benchmark for health services researchers and policy analysts to assess quality of care among patients receiving treatment for mental disorders [28]. MAT represents a minimum threshold of treatment that goes beyond simply making contact with a treatment provider but falls short of meeting the standards for effective treatment as outlined in evidence-based guidelines from organizations such as the American Psychological Association (APA), the National Institute for Health and Care Excellence (NICE), and the World Health Organization (WHO) [29–33]. The most widely adopted definition of MAT, proposed by Wang et al. (2007) [28], defines MAT as receiving either pharmacotherapy, defined as ≥ 1 month of medication plus ≥ four visits to any type of medical doctor, or counseling, defined as ≥ eight visits with any professional. These criteria were informed by recommendations suggesting that at least four visits are generally the minimum necessary for proper medication assessment, initiation, and monitoring during the acute and continuation phases of pharmacotherapy, while eight counseling sessions reflect the minimum shown to be effective in most clinical trials [25]. This definition of MAT builds upon earlier work [34–37] and has since been widely adopted in subsequent research [16, 23–26, 28, 38, 39], although there has been inconsistency in definitions across some studies [27, 40–43].

Despite this variation in definitions, the literature is consistent in showing that many patients who obtain treatment for mental disorders fail to receive MAT. In high-income countries, research suggests that between 20% and no more than 50% of treated individuals meet MAT criteria, while rates in low- and middle-income countries are estimated often to be below 10% [23–25, 28, 44]. This research generally suggests that pharmacotherapy is somewhat more likely to meet MAT criteria than counseling and that, in most cases [40, 43, 45, 46], but not all [47–50], disparities in MAT exist based on socio-economic status, gender, age, and provider type.

The literature on MAT has several important limitations. First, most MAT studies have focused primarily on depression [23–25], with only a few addressing other mental disorders [26, 27, 41, 42]. Second, MAT studies have been carried out much more frequently in high-income than low- or middle-income countries [39, 43, 45, 47]. Third, MAT studies are often based either on small, unrepresentative samples with low response rates [41, 51, 52] or on simulated projections [23] that are made using untenable modeling assumptions [53]. Together, these limitations restrict the generalizability of findings about the prevalence and correlates of MAT and undermine efforts to establish a global understanding of treatment adequacy.

Previous studies carried out by the World Mental Health (WMH) Survey Initiative collaborators sought to describe and analyze the level of care received by patients with mental disorders by leveraging large, cross-national survey datasets to map the mental health treatment cascade for individual disorders by tracing the steps in the help-seeking process from problem recognition to initial contact with the treatment system through receipt of effective care [10, 13, 14]. The current study builds on these earlier studies by using WMH data to estimate prevalence and correlates of 12-month MAT among patients receiving treatment for a range of mental disorders. Understanding these patterns and predictors is crucial for addressing disparities, overcoming barriers, and scaling up effective treatments globally.

## Methods

### Sample

The WMH Survey Initiative is a coordinated series of community epidemiologic surveys carried out in countries around the world using a consistent survey and set of administration methods for purposes of making cross-national comparisons of prevalence and correlates of mental disorders [1, 54]. The current report uses data from 25 WMH surveys carried out in 21 countries between 2001 and 2019, encompassing a total of 117,739 respondents aged 18 and older (Supplementary Table 1, Additional File 1). Sixteen of these surveys were nationally representative, and the other nine were representative of specific regions or metropolitan areas. The participating countries include 13 high-income countries (15 surveys) and eight low-or-middle-income countries (10 surveys), as classified by the World Bank. Response rates varied widely (45.9–97.2%), with a weighted average response rate of 69.3% across surveys, calculated using the American Association for Public Opinion Research’s response rate 1 method for two-phase sample designs [55]. The current report examines data from 1,838 participants across surveys who met the criteria for at least one of nine 12-month disorders and received treatment within 12 months of the survey. Collectively, these participants accounted for 3,538 treated diagnoses, reflecting a high prevalence of comorbid disorders. Person-disorders are the unit of analysis. Treatment was assessed separately for each disorder, so a respondent might be included in the analysis for one disorder they were treated for but excluded from others for which they did not receive treatment. The study follows the Strengthening the Reporting of Observational Studies in Epidemiology (STROBE) guidelines for reporting observational studies [56].

### Study procedures

As detailed elsewhere [57], WMH surveys are carried out by trained lay interviewers who conduct in-person interviews with respondents in their homes. Interviewers undergo standardized training before fieldwork, with measures in place to monitor performance and ensure consistent data collection, cleaning, and coding across surveys. Written informed consent is obtained from all participants before administering interviews. Study procedures are approved by the institutional review boards in each country before surveys are implemented.

To reduce respondent burden, WMH interviews are divided into two parts. Part I, administered to the total sample, assesses core mental disorders. Part II, conducted with a subsample, collects data on additional disorders and correlates, including treatment history. Part II subsample includes all respondents meeting lifetime criteria for any disorder assessed in Part I, along with a probability subsample of other respondents. This Part II sample for the surveys considered here included a total of 56,927 respondents. To account for the under-sampling of Part I respondents in the Part II sample, Part II data were weighted by the inverse probability of selection, ensuring that weighted prevalence estimates for Part I disorders remain consistent between the Part I and Part II samples. Additional weights were applied to adjust for differential probabilities of selection within households and to align the sample with population distributions based on socio-demographic and geographic characteristics from census data. All analyses presented in this study were conducted using the weighted Part II data.

### Measures

#### Socio-demographics

The socio-demographic variables considered in the analysis included sex, age (18–29, 30–44, 45–59, 60 +), education (categorized into four levels based on the country-specific education system) [58]), and marital status (categorized into three levels; married or cohabitating, previously married, and never married).

#### Mental disorders

The WMH surveys use the WHO Composite International Diagnostic Interview (CIDI 3.0), a fully structured interview designed to be administered by trained lay interviewers, to assess mental disorders according to both the Diagnostic and Statistical Manual of Mental Disorders, Fourth Edition (DSM-IV) and the 10th revision of the International Classification of Diseases (ICD-10) criteria [59]. This study focuses on 12-month prevalence of nine disorders that were assessed in common across all the surveys considered here based on DSM-IV criteria: five anxiety disorders (generalized anxiety disorder, panic disorder/agoraphobia, social phobia, specific phobia, and post-traumatic stress disorder), two mood disorders (major depressive disorder and bipolar spectrum disorder, including bipolar I, II, and subthreshold bipolar disorder) [60], and two substance use disorders (alcohol use disorder and drug use disorder, encompassing both abuse and dependence). Previous clinical reappraisal studies have shown that CIDI diagnoses of these disorders have good concordance with diagnoses based on blinded gold standard clinical reappraisal interviews carried out by trained clinical interviewers [61–67].

#### Severity

Each respondent was defined as having a severe, moderate, or mild disorder profile using definitions established in prior WMH studies (e.g., [68–70]). Profiles were defined as severe if the respondent either (i) met criteria for Bipolar I disorder and/or substance use disorder with a physiological dependence syndrome; (ii) made a suicide attempt in the past 12 months; or (iii) reported severe role impairment in any role domain for at least one month in the past 12 due to mental or substance use disorders. If not severe, severity was defined as moderate if the respondent reported moderate role impairment for at least one month or a 12-month substance use disorder without a physiological dependence syndrome. All other cases were defined as mild.

#### Age-of-onset

Participants reported the age when they first experienced symptoms for each disorder. This information was included in the analysis to predict 12-month MAT based on the hypothesis that disorders with an earlier age-of-onset (AOO) may be recognized and treated over time more often than disorders with more recent onset. For each disorder, median and interquartile range (IQR) AOO were also calculated and used to examine associations between AOO and the likelihood of receiving MAT.

#### Perceived need for treatment

Earlier WMH analyses documented a strong association between participants’ recognition of the need for treatment and likelihood of obtaining treatment [13]. However, it remains unclear whether perceived need is also linked to receiving MAT among patients already in treatment. Patients who fail to meet MAT criteria may have sought treatment primarily due to external pressure from others, which might increase the likelihood of treatment dropout. To explore this possibility, perceived need was included as a potential predictor of MAT in the current analysis. Perceived need was operationalized by asking respondents whether seeking professional help was their own decision or if it occurred only due to pressure from others.

#### Treatment sector

WMH respondents were asked if they ever received treatment for issues related to emotions, nerves, mental health, or substance use, and, if so, which types of professionals they ever saw. The categories of providers were adjusted for local contexts while maintaining 11 consistent broad categories across surveys that were subsequently collapsed into four broad categories: (i) *general medical providers:* general practitioner/primary care doctor, any other medical doctor other than a psychiatrist, and any other healthcare provider (e.g., nurse or physician’s assistant other than a mental health provider); (ii) *mental health professionals*: psychiatrist, psychologist, counselor or social workers in a mental health-specialized setting, any other mental health professional (e.g., psychotherapist or mental health nurse); (iii) *human services professionals*: social worker or counselor in a human services setting, spiritual advisor; and *(iv) complementary or alternative medicine (CAM) providers*: internet or self-help groups, or any other type of healer. For each of the 11 broad categories, respondents who reported lifetime treatment were asked about their age when they first sought treatment, recency of treatment, and number of visits in the past 12 months. Separate question series outside the 11 category-specific types were then asked about lifetime receipt of psychotherapy and medication. The lifetime psychotherapy and medication questions did not enquire further about the type of therapy or medication received. However, respondents who reported 12-month use of psychotropic medications were presented with country-specific lists of all available antidepressants, anxiolytics, hypnotics, antipsychotics, and mood stabilizers and asked to report all medications they used, even if only once, over the past 12 months. Importantly, none of the above treatment questions were disorder-specific. A separate set of disorder-specific treatment questions was asked about lifetime treatment, age of first receiving treatment, whether disorder-specific treatments were ever helpful, ages of first receiving helpful disorder-specific treatments, and whether disorder-specific treatments were obtained in the past 12 months. However, none of these disorder-specific questions were sector-specific.

#### Minimally adequate treatment (MAT)

We adopted the definition of MAT established by Wang et al. [28], which, as noted in the introduction, has been widely used in subsequent studies (e.g., [23–26, 28, 38, 39]). Pharmacotherapy was defined as meeting the criteria for MAT if any psychotropic medication was taken for at least one month in the past 12 months in conjunction with four or more visits with a healthcare professional. There was no stipulation that the type of medication had to match the type of diagnosis (e.g., an antidepressant required for major depressive disorder). Counseling was defined as having a psychiatrist visit of more than 30 min, a psychiatrist visit of less than 30 min if no medication was prescribed, or a visit of any length for any provider other than a general medical provider. To meet the criteria for MAT required 8 counseling visits as defined above with any treatment provider other than general medical, but without requiring the provider to be a mental health professional (including CAM providers). All visits and medication use needed to occur within the 12-month period prior to the interview to qualify as MAT. Respondents still in treatment at the time of interview who had not yet made enough visits to qualify as MAT were classified as not meeting criteria for MAT, even though they might have met criteria if we had followed them through their full course of treatment. As detailed below, we disaggregated the analysis of predictors to distinguish failure to meet MAT criteria because of not yet having a sufficient number of visits, versus discontinuing treatment prematurely, versus completing what the respondent referred to as a “full recommended course of treatment” without having enough visits to qualify for MAT.

#### Reasons for discontinuing treatment

We asked participants who were no longer in treatment at the time of interview whether they stopped treatment against the advice of their treatment provider or if they completed the full recommended course of treatment. If the respondent reported stopping against the advice of the treatment provider, we followed up with a series of questions about 14 potential reasons for dropping out of treatment that we subsequently grouped into five categories: (i) *Low perceived severity*: either thinking that the problem would get better on its own, the problem not being very bothersome, and/or wanting to handle the problem on their own; (ii) *financial barriers*: insurance would not pay for treatment, and/or concerns about being able to afford treatment; (iii) *other enabling barriers:* either having problems with things like transportation or scheduling that made it hard to get to treatment, being unsure about where to go or who to see, thinking that treatment would take too much time or be inconvenient, and/or not being able to get an appointment; (iv) *low perceived treatment effectiveness*: either not being satisfied with available treatments, not thinking treatment will work, and/or getting treatment in the past and not finding it helpful; and (v) *perceived stigma*: concern about what people would think if they found out the patient was in treatment and/or worried about being involuntarily committed to a hospital.

### Statistical analysis procedures

Cross-tabulations were used to estimate 12-month MAT prevalence among individuals with disorders who received treatment. Multivariable regression analysis was then used to investigate associations with MAT. We began by considering predictors one at a time, then grouped them into broad classes (socio-demographics, disorder characteristics, prior treatment history, and current treatment characteristics), and finally, considering all significant within-class predictors in a single model.

After estimating the final model, we disaggregated the associations of the significant predictors with MAT to look separately at predictors of failure due to (i) discontinuing treatment against the advice of the treatment provider, (ii) completing a full course of treatment that did not meet MAT criteria, or (iii) still being in treatment at the time of survey but not yet meeting criteria for MAT. In addition, for patients who discontinued treatment, we examined the role of the five broad categories of reported reasons in explaining associations of significant predictors with MAT. The latter analysis involved a special type of subgroup comparison in which we compared predictors of MAT versus premature discontinuation in the total sample and then again in subsamples that sequentially excluded respondents reporting a specific type of barrier. Differences in predictor coefficients between the restricted samples and the total sample were used to infer the importance of the excluded barrier. This indirect type of inference was used rather than a more conventional control variable analysis because conventional control variable analysis is not possible when none of the people with the control variables (i.e., those who reported barriers) experienced the dependent variable (i.e., received MAT).

All regression models used a Poisson link function with robust standard errors that accounted for the weighting and geographic clustering in the WMH surveys [71]. The regression coefficients were exponentiated to derive risk ratios (RRs), while 95% confidence intervals were calculated as the regression coefficients ± 2 design-based standard errors. The significance of categorical predictors was evaluated using Wald χ^2^ tests based on design-adjusted variance–covariance matrices. Statistical significance was evaluated using two-sided design-based 0.05-level tests.

## Results

### Sample characteristics

As noted above, the sample included n = 3,538 12-month treated disorders reported by n = 1,838 respondents. Two-thirds (68.8%) of these disorders occurred to women, 69.4% to respondents aged 30–59, 42.8% to respondents with low or low-average education, and 49.9% to those who were married (Supplementary Table 2, Additional File 1). Associations between these sample characteristics and disorder prevalence [10], perceived need for treatment given prevalence [72], and obtaining any treatment given prevalence in the presence and absence of perceived need [13] have been discussed in previous reports. It is noteworthy that treated 12-month disorders represent 17.7% of all 12-month disorders. That is, 82.3% of 12-month disorders were not treated, with only modest variation in this proportion across countries [13].

###  Disorder characteristics

Approximately two-thirds (66.2%) of treated disorders met MAT criteria, with only modest variation across disorders (62.3–72.1%) (Table 1). Greater variation was observed based on the respondent’s number of disorders (55.3–73.7%) and disorder severity (57.1–73.4%). Regression analysis showed no significant variation in MAT prevalence among treated cases by disorder type (χ^2^_8_ = 11.4, p = 0.19) or years since disorder onset (median: 16 years, IQR: 5–30) (χ^2^_1_ = 0.2, p = 0.63) in the univariable analysis (Table 2). The number of 12-month disorders for which the respondent met criteria was associated with increased probability of MAT in a univariable model (χ^2^_2_ = 10.2, p < 0.001) but not in a multivariable within-domain model (χ^2^_2_ = 0.1, p = 0.97). A multivariable disorder profile developed in an earlier WMH report to predict effective treatment in both treated and untreated cases [10] was a significant predictor of MAT not only in the univariable (χ^2^_1_ = 41.8, p < 0.001) and within-domain multivariable (χ^2^_1_ = 17.8, p < 0.001) models, but also in a preliminary consolidated model that included all initially significant within-domain predictors across predictor domains (χ^2^_1_ = 4.0, p = 0.048). Disorder severity, finally, was a significant predictor in all models (χ^2^_2_ = 35.0–6.8, p < 0.001–0.035), with the RR of MAT significantly higher for severe (RR = 1.3) than moderate (RR = 0.9) or mild (RR = 1.0) cases in the univariable model but RR differing only modestly by severity in the final model (RR = 0.9–1.0).

**Table 1:** Prevalence of MAT by 12-month DSM/CIDI disorders among treated cases (n = 3,538)^a^

	Disorder prevalence among treated cases			MAT among treated cases	
I. Anxiety	**n**	**%**	**(SE)**	**%**	**(SE)**
GAD	516	14.5	(0.5)	69.4	(2.0)
Panic/Ago	459	13.0	(0.5)	68.6	(2.3)
PTSD	327	9.3	(0.5)	65.8	(3.0)
Specific	535	14.0	(0.5)	66.7	(2.6)
Social	414	11.9	(0.4)	66.1	(3.5)
Any	2251	62.6	(0.7)	67.5	(1.9)
II. Mood disorders					
MDD	999	28.3	(0.8)	62.3	(1.6)
BD	167	4.7	(0.4)	72.1	(3.7)
Any	1166	32.9	(0.7)	63.7	(1.4)
III. Substance use disorder					
AUD	83	3.1	(0.3)	66.9	(6.1)
DUD	38	1.4	(0.3)	69.0	(9.3)
Any	121	4.4	(0.5)	67.6	(6.3)
IV. Number of disorders					
1	756	22.0	(1.0)	55.3	(1.9)
2	1057	29.6	(1.2)	62.2	(2.3)
3 +	1725	48.3	(1.5)	73.7	(2.6)
V. Severity					
Severe	2105	60.7	(1.4)	73.4	(1.7)
Moderate	1152	31.1	(1.3)	54.6	(2.6)
Mild	281	8.2	(0.8)	57.1	(3.6)
Any disorder	3538	100.0	(0.0)	66.2	(1.5)
(n) person disorder	(3,538)			(3,538)	
(n) person level	(1,838)			(1,838)	

*MAT* Minimally adequate treatment; *%* proportion of observation in the column or row total; *SE* the design-based standard error of % taking into consideration the weighting and geographic clustering of observations; *GAD* generalized anxiety disorder; *Panic/AGO* panic disorder or agoraphobia; *PTSD* post-traumatic stress disorder; *Specific* specific phobia; *Social* social phobia; *MDD* major depressive disorder; *BD* bipolar spectrum disorder; *AUD* alcohol use disorder (either abuse or dependence); *DUD* drug use disorder (either abuse or dependence); *Severe* the subset of respondents with either 12-month BD, AUD with a physiological dependence syndrome, DUD with a physiological dependence syndrome, suicide attempt, of self-reported severe role impairment due to their 12-month mental and/or substance use disorders. *Moderate* the subset of respondents without severe disorder who reported moderate role impairment due to their 12-month mental and/or substance use disorders; *Mild* the subset of respondents with a 12-month disorder who do not qualify for either severe or moderate disorder; *Any* entries in the Any rows are the weighted averages of the entries in the above rows within the same subset; *(n) person disorder* the unweighted number of survey observations in the denominator (i.e., in the subsample of Part II 12-month person-disorders with treatment contact); *(n) person level* the unweighted number of survey observations in the denominator (i.e., in the subsample of Part II f person-level survey respondents with a 12-month disorder who had any treatment); % of Disorder prevalence represents the proportion of observations in the column; % of the MAT among treated cases is among the disorders indicated in the row headers

^a^Pooled across all WMH surveys, with surveys weighted by sample size rather than by country population size

**Table 2:** Pooled within-country associations of disorder characteristics with MAT among treated cases (n = 3,538)^a^

	Distribution		Univariable		Multivariable		Preliminary consolidated multivariable	
	%	(SE)	RR	(95% CI)	RR	(95% CI)	RR	(95% CI)
I. Multivariate disorder profile for effective treatment	-	-	1.1*	(1.0–1.1)	1.1*	(1.0–1.1)	1.0*	(1.0–1.0)
* χ*^2^_1_	-		41.8*		17.8*		4.0*	
II. Anxiety disorders^b^								
GAD	14.5	(0.5)	1.0	(1.0–1.1)	1.0	(0.9–1.1)	-	-
Panic/Ago	13.0	(0.5)	1.0	(0.9–1.1)	1.0	(0.9–1.0)	-	-
PTSD	9.3	(0.5)	1.0	(0.9–1.0)	0.9	(0.9–1.0)	-	-
Specific	14.0	(0.5)	1.0	(0.9–1.1)	1.0	(1.0–1.1)	-	-
Social	11.9	(0.4)	1.0	(0.9–1.1)	0.9	(0.9–1.0)	-	-
III. Mood disorders								
MDD	28.3	(0.8)	0.9	(0.9–1.0)	1.0	(0.9–1.1)	-	-
BD	4.7	(0.4)	1.1	(1.0–1.2)	1.1	(1.0–1.2)	-	-
IV. Substance use disorders								
AUD	3.1	(0.3)	1.0	(0.9–1.2)	1.0	(0.9–1.2)	-	-
DUD	1.4	(0.3)	1.0	(0.8–1.3)	1.0	(0.7–1.3)	-	-
* χ*^2^_8_	-		11.4		11.0		-	
V. Number of disorders								
1	22.0	(1.0)	1.0	-	-	-	-	-
2	29.6	(1.2)	1.1	(1.0–1.2)	1.0	(0.9–1.2)	-	-
3 +	48.3	(1.5)	1.3*	(1.1–1.4)	1.0	(0.9–1.2)	-	-
*χ*^2^_2_	-		10.2*		0.1		-	
VI. Severity								
Severe	22.0	(1.0)	1.3*	(1.1–1.5)	1.1	(1.0–1.3)	1.0	(0.9–1.1)
Moderate	29.6	(1.2)	0.9	(0.8–1.1)	0.9	(0.8–1.0)	0.9	(0.8–1.0)
Mild	48.3	(1.5)	1.0	-	1.0	-	1.0	-
*χ*^2^_2_	-		35.0*		18.1*		6.8*	
VII. Number of years since dx onset								
Continuous (standardized)^c^	16	(5,30)	1.0	(1.0–1.0)	1.0	(1.0–1.0)	-	-
*χ*^2^_1_	-		0.2		1.0		-	

*Univariable* associations of each row predictor with minimally adequate treatment in a separate model controlling only survey; *Multivariable* associations of all disorder-related predictors with minimally adequate treatment in a single model controlling for survey*; Preliminary consolidated multivariable* associations of all predictors disorder-related predictors with minimally adequate treatment in a single model controlling for survey and predictors involving prior and 12-month treatment history; *RR* relative risk of minimally adequate treatment as a function of the row predictor; *95% CI* the design-based 95% confidence interval of RR, taking into consideration the weighting and geographic clustering of observations; *GAD* generalized anxiety disorder; *Panic/AGO* panic disorder or agoraphobia; *PTSD* post-traumatic stress disorder; *Specific* specific phobia; *Social* social phobia; *MDD* major depressive disorder; *BD* bipolar spectrum disorder; *AUD* alcohol use disorder (either abuse or dependence);* DUD*, drug use disorder (either abuse or dependence); *Severe* the subset of respondents with either 12-month BD, AUD with a physiological dependence syndrome, DUD with a physiological dependence syndrome, suicide attempt, of self-reported severe role impairment due to their 12-month mental and/or substance use disorders; *Moderate* the subset of respondents without severe disorder who reported moderate role impairment due to their 12-month mental and/or substance use disorders; *Mild* the subset of respondents with a 12-month disorder who do not qualify for either severe or moderate disorder; *dx* diagnosis

^a^Pooled across all WMH surveys, with surveys weighted by sample size rather than by country population size

^b^In the multivariate and univariate model for type of disorder, RRs for type of disorder were scaled so that the product of the RRs was 1.0 across disorders

^c^The mean and standard error number of years since onset of the disorder were 19.1 and 0.3 respectively. The variable was standardized to a mean of 0 and standard deviation of 1 for purposes of analysis

### Socio-demographic predictors

There was no significant socio-demographic variation in MAT among treated cases, with respondent sex (*χ*^2^_1_ = 0.5, p = 0.48), age (*χ*^2^_3_ = 0.3, p = 0.96), education (*χ*^2^_3_ = 3.6, p = 0.32), marital status (*χ*^2^_2_ = 5.1, p = 0.08), and perceived need (*χ*^2^_1_ = 0.7, p = 0.41) all nonsignificant in univariable models (Supplementary Table 2, Additional File 1).

### Prior treatment history

We next examined the associations of prior (pre-12 months) lifetime treatment with 12-month MAT among treated cases. Most (80.8%) respondents with 12-month treatment had a history of prior treatment (Supplementary Table 3, Additional File 1). Type of prior treatment provider significantly predicted 12-month MAT in a univariable model (*χ*^2^_5_ = 52.9, p < 0.001), with modestly elevated relative risks (RR = 1.2–1.3) observed for prior treatment by psychiatrists and other mental health professionals, compared to other providers (RR = 0.9–1.0). This association persisted in the within-domain multivariable model (*χ*^2^_5_ = 34.2, p < 0.001), but became nonsignificant in the preliminary consolidated model (*χ*^2^_5_ = 6.1, p = 0.31). The same general pattern held for perceived helpfulness of prior treatment, which was significant in both the univariable (*χ*^2^_3_ = 8.3, p = 0.042) and multivariable (*χ*^2^_2_ = 6.6, p = 0.038) models, but became nonsignificant in the preliminary consolidated model (*χ*^2^_2_ = 1.5, p = 0.48). The other aspects of prior treatment considered – number of prior types of providers seen and types of treatment received (i.e., medication-only, psychotherapy-only, or both) – were significant in univariable models (*χ*^2^_5/2_ = 24.8–20.9, p < 0.001) but not in the multivariable model (*χ*^2^_4/2_ = 6.8–4.7, p = 0.15–0.10). The net result was that no aspect of prior lifetime treatment remained in the consolidated model.

### Current treatment characteristics

Characteristics of 12-month treatment emerged as more important predictors of MAT (Table 3), with significant univariable associations observed for both provider type (RR = 1.2–1.6; χ^2^_5_ = 272.8, p < 0.001) and number of provider types (RR = 1.8–2.3; *χ*^2^_4_ = 189.0, p < 0.001). Treatment by psychiatrists and other mental health professionals was associated with the highest relative risks (RR = 1.6) compared to other provider types (RR = 1.0–1.2) in the univariable model for provider type. Provider type remained significant in the within-domain multivariable and preliminary consolidated models (χ^2^_5_ = 133.2–95.9, p < 0.001), but significant associations were observed for all provider types in both these models (RR = 1.3–1.8), indicating that treatment by any two or more provider types was significantly associated with a higher likelihood of receiving MAT compared to treatment by only one provider type. Furthermore, the relative risks associated with number of provider types exceeding two *decreased* in both the within-domain multivariable and preliminary consolidated models (RR = 0.7–0.3; χ^2^₃ = 25.9–29.0, p < 0.001) despite monotonically *increasing* RRs in the univariable model (RR = 1.8–2.3; χ^2^₃ = 189.0, p < 0.001). This inverse association does not imply that being treated by three or more provider types is worse than being treated by two; rather, it suggests that the positive association between additional provider types and MAT diminishes as the number of provider types increases. Finally, the type of 12-month treatment received – either medication-only, counseling-only, or combined medication-counseling – was a significant predictor in all models (χ^2^_2_ = 127.4–14.7, p < 0.001) due to a somewhat higher RR of MAT with combined treatment (RR = 1.4–1.1) than either medication-only (RR = 0.7–1.0) or counseling-only (RR = 0.9). These RRs were derived using ipsative coding, ensuring that the product of the three RRs equals 1.0 and allowing the RR for each type of treatment to be interpreted relative to the other types of treatment. Coefficient estimates remained largely consistent between univariable and multivariable models, indicating minimal issues with multicollinearity among predictors.

**Table 3:** Pooled within-country associations of 12-month treatment characteristics with MAT among treated cases (n = 3,538)^a^

	Distribution		Univariable		Multivariable		Preliminary consolidated multivariable	
	%	(SE)	RR	(95% CI)	RR	(95% CI)	RR	(95% CI)
I. 12-month provider type								
Psychiatrist	52.2	(1.5)	1.6*	(1.5–1.8)	1.8*	(1.6–2.1)	1.8*	(1.6–2.1)
Other mental health	45.6	(1.6)	1.6*	(1.5–1.8)	1.8*	(1.6–2.1)	1.8*	(1.5–2.1)
General medical	56.1	(1.3)	1.1	(1.0–1.2)	1.3*	(1.1–1.5)	1.3*	(1.1–1.5)
Human services	12.2	(1.0)	1.0	(0.9–1.1)	1.3*	(1.1–1.6)	1.4*	(1.2–1.7)
CAM	13.8	(1.1)	1.2*	(1.1–1.3)	1.5*	(1.3–1.8)	1.6*	(1.3–1.9)
χ^2^_5_	-		272.8*		133.2*		95.9*	
II. Number of 12-month provider types								
1	48.7	(1.5)	1.0	-	-	-	-	-
2	29.8	(1.3)	1.8*	(1.6–2.0)	1.0	-	1.0	-
3	15.7	(0.9)	2.1*	(1.9–2.4)	0.7*	(0.6–0.9)	0.7*	(0.6–0.9)
4	4.3	(0.6)	2.2*	(1.9–2.5)	0.5*	(0.4–0.7)	0.5*	(0.4–0.7)
5	1.5	(0.6)	2.3*	(2.0–2.6)	0.4*	(0.2–0.5)	0.3*	(0.2–0.5)
χ^2^_4/3_	-		189.0*		25.9*		29.0*	
III. 12-month treatment types^b^								
Medication-only	29.5	(0.1)	0.7*	(0.7–0.8)	1.0	(0.9–1.1)	1.0	(0.9–1.1)
Counseling-only^c^	16.8	(0.1)	0.9	(0.9–1.0)	0.9*	(0.8–1.0)	0.9*	(0.8–1.0)
Combined	53.6	(0.2)	1.4*	(1.3–1.5)	1.1*	(1.1–1.2)	1.1*	(1.0–1.2)
χ^2^_2_	-		127.4*		19.1*		14.7*	
χ^2^_1_	-		7.2*		1.7		1.4	

*Univariable* associations of each row predictor with minimally adequate treatment in a separate model controlling only survey; *Multivariable* associations of all 12 month provider predictors with minimally adequate treatment in a single model controlling for survey; *Preliminary consolidated multivariable* associations of all 12-month treatment history predictors with minimally adequate treatment in a single model controlling for survey and disorder-related predictors and prior treatment history predictors RR, relative risk of minimally adequate treatment as a function of the row predictor; *95% CI* the design-based 95% confidence interval of RR, taking into consideration the weighting and geographic clustering of observations

^a^Pooled across all WMH surveys, with surveys weighted by sample size rather than by country population size

^b^In the univariate model for 12-month treatment types and the multivariate model, RRs for 12-month treatment types were scaled so that the product of the RRs was 1.0 across types

^c^Counseling was defined as having a 30-min or longer visit with any of their 12 month service providers

^*^Significant at the.05 level, two-sided design-based test

### Disaggregating failure to receive minimally adequate treatment

Participants failing to receive MAT were categorized into three groups: (i) those who discontinued treatment against the advice of the treatment provider (n = 231), (ii) those who completed a full recommended course of treatment that did not meet MAT criteria (n = 180), and (iii) those who were still in treatment but had too few visits at the time of the interview to qualify for MAT (n = 814). We then estimated a series of models containing the final consolidated set of significant predictors to determine which of these predictors was associated with each of these three ways of failing to meet MAT criteria (Table 4). The multivariable disorder profile remained significant in the MAT vs. premature discontinuation (χ^2^_1_ = 6.3, p = 0.014) and MAT vs. still in treatment (χ^2^_1_ = 4.1, p = 0.045) models, but not in the MAT vs. completed treatment model (χ^2^_1_ = 0.2, p = 0.70), suggesting that the overall association between the disorder profile and MAT was largely due to the disorder profile predicting a low probability of receiving an inadequate course of treatment. In contrast, disorder severity followed the opposite pattern, remaining significant in the MAT vs. completed treatment model (χ^2^_2_ = 11.8, p = 0.003) but not in the other two models (χ^2^_2_ = 3.0–2.9, p = 0.22–0.24), suggesting that the modest overall association between disorder severity and MAT was largely due to disorder severity predicting a low probability of both discontinuing treatment and remaining in treatment at the time of interview.

**Table 4:** Pooled within-country predictors of MAT disaggregated by type of failure to meet criteria for MAT^a^

	Consolidated multivariable in total sample		MAT vs. premature discontinuation		MAT vs. completed treatment		MAT vs. still in treatment	
	RR	(95% CI)	RR	(95% CI)	RR	(95% CI)	RR	(95% CI)
I. Multivariate disorder profile for effective treatment	1.0*	(1.0–1.1)	1.0*	(1.0–1.0)	1.0	(1.0–1.0)	1.0*	(1.0–1.0)
χ^2^_1_	5.3*		6.3*		0.2		4.1*	
II. Severity								
Severe	1.0	(0.9–1.1)	0.9	(0.9–1.0)	1.0	(1.0–1.1)	1.0	(0.9–1.1)
Moderate	0.9	(0.8–1.0)	0.9	(0.9–1.0)	1.0	(0.9–1.0)	0.9	(0.8–1.0)
χ^2^_2_	6.8*		3.0		11.8*		2.9	
III. Types of 12-month providers								
Psychiatrist	1.8*	(1.6–2.1)	1.2*	(1.1–1.3)	1.2*	(1.1–1.2)	1.6*	(1.4–1.8)
Other mental health	1.8*	(1.6–2.1)	1.2*	(1.1–1.3)	1.2*	(1.1–1.2)	1.6*	(1.4–1.8)
General medical	1.3*	(1.1–1.5)	1.1*	(1.0–1.2)	1.1*	(1.0–1.1)	1.2*	(1.0–1.3)
Human services	1.3*	(1.1–1.6)	1.2*	(1.1–1.2)	1.1*	(1.1–1.2)	1.2	(1.0–1.4)
CAM	1.5*	(1.3–1.8)	1.2*	(1.1–1.3)	1.2*	(1.1–1.2)	1.3*	(1.1–1.5)
χ^2^_5_	127.7*		35.0*		44.5*		97.2*	
IV. Number of 12-month provider types								
3	0.7*	(0.6–0.9)	0.9*	(0.8–0.9)	0.9*	(0.8–0.9)	0.8*	(0.7–0.9)
4	0.5*	(0.4–0.7)	0.8*	(0.7–0.9)	0.8*	(0.7–0.9)	0.6*	(0.5–0.8)
5	0.3*	(0.2–0.5)	0.6*	(0.5–0.8)	0.7*	(0.6–0.8)	0.5*	(0.3–0.7)
χ^2^_3_	32.5*		31.3*		30.8*		12.9*	
V. 12-month treatment types^b^								
Medication-only	1.0	(0.9–1.1)	1.0	(1.0–1.1)	1.0	(1.0–1.0)	1.0	(0.9–1.1)
Counseling-only^c^	0.9*	(0.8–1.0)	0.9	(0.9–1.0)	1.0	(0.9–1.0)	1.0	(0.9–1.0)
Combined	1.1*	(1.0–1.2)	1.0*	(1.0–1.1)	1.0	(1.0–1.1)	1.1*	(1.0–1.2)
χ^2^_2_	14.8*		5.8		2.2		7.5*	
(n)	(3,538)		(2,544)		(2,493)		(3,127)	

*Consolidated multivariable in total sample* total sample of treated respondents either with or without MAT; *MAT vs premature discontinuation* respondents with MAT (Coded 1 on the outcome) or without MAT due to discontinuing treatment (Coded 0); *MAT vs completed treatment* respondents with MAT (Coded 1 on the outcome) or without MAT after completing the recommended course of treatment; *MAT vs still in treatment* respondents with MAT (Coded 1 on the outcome) or still in treatment but not qualifying for MAT at time of interview; *RR* risk-ratio; *95% CI* design-based 95% confidence interval of RR taking into consideration the weighting and geographic clustering of the WMH data; *n* number of observations in the comparison

^a^Based on multivariable Poisson regression models to predict 12-month MAT (Coded 1 on the outcome) versus not MAT (Coded 0 on the outcome) across all WMH surveys, with surveys weighted by sample size rather than by country population size with dummy variables for country included as controls, allowing coefficients to be interpreted as pooled weighted within-country coefficients

^b^In the univariable model for 12-month treatment types and the multivariable model, RRs for 12-month treatment types were scaled so that the product of the RRs was 1.0 across types

^c^Counseling was defined as having a 30-min or longer visit with any of their 12 month service providers

^*^Significant at the.05 level, two-sided design-based test

Type of provider remained a significant predictor in each disaggregated model (χ^2^_5_ = 35.0–97.2, p < 0.001), but with much weaker associations in the MAT vs. premature discontinuation and MAT vs. completed treatment models (RR = 1.1–1.2) than in the MAT vs. still in treatment model (RR = 1.2–1.6), indicating joint influences across all mediators in accounting for the more substantial overall associations (RR = 1.3–1.8). A similar pattern was found for the number of types of treatment providers, which was significant in each disaggregated model (χ^2^_3_ = 31.3–12.9, p < 0.001–0.006), indicating joint influences across all mediators. Finally, treatment type remained significant in the MAT vs. still in treatment model (χ^2^_2_ = 7.5, p = 0.024) but became nonsignificant in the other disaggregated models (χ^2^_2_ = 5.8–2.2, p = 0.06–0.34), suggesting that the modest overall association of treatment type with MAT was due to low probability of remaining in non-MAT treatment at the time of interview.

### Reasons for discontinuing treatment

The most common reason for discontinuing treatment was low perceived disorder severity (69.5%), even though our analysis of disorder-related predictors found that objective disorder severity was only associated weakly with MAT (Supplementary Table 4, Additional File 1). The second most common reason was low perceived treatment effectiveness (42.1%), followed by financial barriers (22.3%), other enabling barriers (17.5%), and perceived stigma (14.8%). Over half (50.3%) of the respondents who discontinued treatment prematurely cited a single reason for doing so, while 20.2% mentioned two reasons, and 23.8% cited three or more reasons.

We investigated the extent to which these reported reasons explained the associations between significant predictors and failure to receive MAT due to treatment discontinuation (Table 5). The base associations (which differed somewhat from those in Table 4 due to Table 5 excluding the n = 32 cases of premature discontinuation where the survey respondent failed to report any reasons for discontinuation) were all quite modest in magnitude (RR = 0.7–1.2), making it difficult to detect variation across comparisons. However, inspection of χ^2^ values showed that the significant association of the disorder profile with premature discontinuation disappeared when we excluded either the respondents who reported low perceived treatment effectiveness or perceived stigma as reasons for discontinuation. The situation was different for types and number of providers, which remained significant in each comparison, but with χ^2^ values that were lowest when we excluded respondents who reported low perceived severity as a reason for discontinuation. The association of type of treatment with premature discontinuation, finally, which was significant here although not in Table 4, was accounted for by each of the reasons other than financial barriers.

**Table 5:** Pooled within-country predictors of MAT mediated through barriers^a^

			Subgroup models excluding barriers in the column headings													
	MAT vs premature discontinuation		Low perceived severity			Financial			Other enabling			Low perceived treatment effectiveness			Perceived stigma	
	RR	(95% CI)	RR	(95% CI)		RR	(95% CI)		RR	(95% CI)		RR	(95% CI)		RR	(95% CI)
I. Multivariate disorder profile for effective treatment	1.0*	(1.0–1.0)	1.0*	(1.0–1.0)		1.0*	(1.0–1.0)		1.0*	(1.0–1.0)		1.0	(1.0–1.0)		1.0	(1.0–1.0)
χ^2^_1_	4.7*		5.0*			8.2*			4.3*			2.1			3.8	
I. Severity																
Severe	0.9	(0.9–1.0)	1.0	(1.0–1.1)		1.0	(0.9–1.0)		1.0	(0.9–1.0)		1.0	(0.9–1.0)		1.0	(0.9–1.0)
Moderate	0.9*	(0.9–1.0)	1.0	(1.0–1.1)		1.0	(0.9–1.0)		0.9	(0.9–1.0)		1.0	(0.9–1.0)		1.0	(0.9–1.0)
χ^2^_2_	4.3		2.3			1.9			3.1			1.8			2.3	
III. Types of 12-month providers																
Psychiatrist	1.2*	(1.1–1.3)	1.1*	(1.0–1.1)		1.1*	(1.1–1.2)		1.1*	(1.1–1.2)		1.1*	(1.0–1.2)		1.2*	(1.1–1.2)
Other mental health	1.2*	(1.1–1.3)	1.0*	(1.0–1.1)		1.2*	(1.1–1.2)		1.2*	(1.1–1.3)		1.1*	(1.0–1.2)		1.1*	(1.1–1.2)
General medical	1.1*	(1.0–1.2)	1.0*	(1.0–1.1)		1.1*	(1.0–1.1)		1.1	(1.0–1.1)		1.0	(1.0–1.1)		1.1*	(1.0–1.1)
Human services	1.1*	(1.1–1.2)	1.0	(1.0–1.1)		1.1*	(1.0–1.2)		1.1*	(1.0–1.2)		1.1*	(1.0–1.1)		1.1*	(1.1–1.2)
CAM	1.2*	(1.1–1.2)	1.0*	(1.0–1.1)		1.1*	(1.1–1.2)		1.1*	(1.1–1.2)		1.1*	(1.1–1.2)		1.2*	(1.1–1.2)
χ^2^_5_	35.0*		13.3*			32.9*			26.9*			26.3*			34.5*	
IV. Number of 12-month provider types																
3	0.9*	(0.8–1.0)	1.0*	(0.9–1.0)		0.9*	(0.9–1.0)		0.9*	(0.9–1.0)		0.9*	(0.9–1.0)		0.9*	(0.9–1.0)
4	0.8*	(0.7–0.9)	0.9*	(0.9–1.0)		0.8*	(0.7–0.9)		0.8*	(0.7–0.9)		0.9*	(0.8–1.0)		0.8*	(0.7–0.9)
5	0.7*	(0.6–0.8)	0.9*	(0.8–0.9)		0.7*	(0.6–0.8)		0.7*	(0.6–0.9)		0.8*	(0.7–0.9)		0.7*	(0.6–0.8)
χ^2^_3_	31.1*		14.1*			21.0*			23.8*			21.4*			35.0*	
V. 12-month treatment types^b^																
Medication-only	1.0	(1.0–1.1)	1.0	(1.0–1.0)		1.0	(1.0–1.1)		1.0	(1.0–1.1)		1.0	(1.0–1.0)		1.0	(1.0–1.1)
Counselingy-only^c^	0.9*	(0.9–1.0)	1.0	(1.0–1.0)		0.9*	(0.9–1.0)		0.9*	(0.9–1.0)		1.0	(0.9–1.0)		1.0	(0.9–1.0)
Combined	1.0	(1.0–1.1)	1.0	(1.0–1.0)		1.0*	(1.0–1.1)		1.0	(1.0–1.0)		1.0	(1.0–1.0)		1.0	(1.0–1.0)
χ^2^_2_	6.2*		0.5			7.6*			4.6			1.6			1.5	
(n)	(2,512)		(2,377)			(2,472)			(2,465)			(2,439)			(2,495)	

*RR* risk-ratio predicting 12-month minimally adequate treatment (coded 1) versus those without minimally adequate, or those without minimally adequate only due to quitting treatment early on after the first model, (coded 0) based on a multivariable regression model using a Poisson link function for pooled within-country associations of the predictors with the outcome; *95% CI* design-based 95% confidence interval of RR taking into consideration the weighting and geographic clustering of the WMH data; *n* the number of observations in the MAT vs. premature discontinuation sample excluding those that reported the reason for discontinuation reported in the column heading

^a^Based on multivariable Poisson regression models to predict 12-month minimally adequate treatment (coded 1) compared to those without minimally adequate treatment (coded 0) across all WMH surveys, with surveys weighted by sample size rather than by country population size with dummy variables for country included as controls, allowing coefficients to be interpreted as pooled weighted within-country coefficients

^b^In the univariate model for 12-month treatment types and the multivariate model, RRs for 12-month treatment types were scaled so that the product of the RRs was 1.0 across types

^c^Counseling was defined as having a 30-min or longer visit with any of their 12 month service providers

^*^Significant at the.05 level, two-sided design-based test

## Discussion

This cross-national study provides an examination of minimally adequate treatment patterns and predictors across a diverse sample from the WMH surveys. Approximately two-thirds of 12-month treated cases met MAT criteria, highlighting persistent gaps in treatment adequacy worldwide. The likelihood of receiving MAT was not significantly associated with disorder type, number of disorders, or years since disorder onset, although it was associated modestly with a multivariate disorder profile and disorder severity. Socio-demographic predictors and prior lifetime treatment were not significant predictors. However, 12-month treatment characteristics were more important, most notably receiving care from mental health specialists and multiple provider types. These findings highlight the importance of treatment accessibility and provider diversity in ensuring adequate care.

Among those who failed to receive MAT, nearly equal proportions discontinued treatment prematurely and completed a full course of inadequate treatment. We found some variation in the importance of predictors across types of failure to receive MAT, the most notable being that provider type was more strongly associated with still being in non-MAT treatment at the time of interview than with either discontinuing treatment or completing a full course of inadequate treatment, although provider type was associated significantly with all three of these types of failure to receive MAT. Further, patients who completed a full course of treatment that did not qualify as MAT were disproportionately treated by a single provider type rather than multiple providers but did not differ from those receiving MAT in terms of treatment modality. This suggests that treatment integration across multiple providers may be more important than the specific treatment approach in determining whether patients receive minimally adequate care. To the extent that the predictors were associated with discontinuation, finally, a variety of reasons emerged as important, although low perceived disorder severity was the most important mediator of the strongest overall predictors (i.e., type and number of providers seen).

Our results also align with other studies highlighting global deficiencies in the adequacy of treatment of common mental disorders [23, 24, 26, 27]. It is noteworthy, though, that our MAT prevalence is higher than previously reported estimates [23–26, 28, 38, 39]. This difference reflects our focus exclusively on treated cases rather than calculating MAT prevalence among all individuals with disorders and variations in MAT definitions across studies, with some using more stringent criteria than those employed in this study. Further, and as noted in the initial paper in this series [10], only a minority of patients (47%) who meet MAT criteria receive treatment that is fully consistent with evidence-based guidelines. This distinction between minimally adequate treatment and fully guideline-concordant care is important for properly understanding the treatment quality continuum. While MAT represents an important minimum threshold of treatment adequacy, it does not necessarily indicate optimal care. Forthcoming analyses will examine factors associated with this transition from minimally adequate to guideline-concordant treatment, representing a critical next step in addressing gaps in the mental health treatment cascade.

Overall, these results underscore the need to educate patients about disorder severity to reduce premature treatment discontinuation. Potential interventions could include psychoeducation at treatment initiation for both patients and their family members or support networks, implementation of standardized severity assessments with regular feedback to patients, integration of digital self-monitoring tools that complement and augment traditional treatments, and training providers in motivational interviewing techniques to address ambivalence about continuing treatment [73–79].

Previous WMH analyses have examined different stages of the mental health treatment cascade, providing context for our current findings. Viana et al. (2025) investigated barriers to initial treatment access, finding that low perceived severity was the primary reason individuals did not seek treatment—mirroring our finding that the same factor drives premature discontinuation among those who begin treatment. Stein et al. (2025) demonstrated that treatment contact was primarily determined by disorder characteristics, health insurance, and employment status. In contrast, our results show that once patients enter treatment, these socio-demographic factors become less influential for treatment adequacy, with provider characteristics emerging as the key determinants. Finally, Vigo et al. (2025) examined the entire treatment pathway to effective care, finding multiple bottlenecks, including low perceived need and ineffective treatment despite adequacy. This evolving pattern of predictors across the treatment continuum highlights the shifting nature of barriers at different stages of care. Specifically, our results show that treatment provided by mental health specialists and multiple provider types were the most important predictors of receiving MAT, whereas socio-demographic factors were not significant – a finding consistent with earlier research [47–50]. It is also noteworthy that an earlier WMH report showed that cross-national indicators of differences in access to treatment, including the number of mental health specialists per capita and national healthcare expenditures, were not significant predictors of between-country differences in MAT when adjusting for compositional differences in individual-level predictors [10]. This suggests that increasing overall health spending alone may not be sufficient to improve treatment adequacy unless resources are strategically allocated to improve provider capacity and treatment quality. While our analysis focused on pooled within-country associations to identify common individual-level predictors across contexts, we acknowledge the importance of understanding how these relationships might vary across different healthcare systems and socioeconomic contexts. Future research should examine how country-specific factors (e.g., healthcare financing mechanisms, provider training standards, cultural attitudes toward mental health) might moderate the relationships we observed. Multilevel analyses that explicitly model country-level characteristics and their interactions with individual-level predictors would be particularly valuable for developing context-sensitive approaches to improving treatment adequacy globally.

The study had several limitations. First, our MAT definition, though widely used, represents a minimum threshold of treatment adequacy rather than a gold standard for effective care. Although this makes MAT a pragmatic benchmark, it does not fully capture whether treatment is clinically optimal or adheres to evidence-based guidelines. Nonetheless, prior research has shown that meeting MAT criteria (as defined in this study) is associated with significant symptom improvements [43], making it a reasonable target for research as well as monitoring and evaluation in real-world settings. Second, the study’s cross-sectional design prevents clear inferences about temporal order between predictors, mediators, and MAT, although most of the assumed temporal orders have strong face validity. Third, the scope of the variables examined was limited. While we explored patient characteristics and treatment-related factors, we could not assess the influence of provider characteristics or health system-level factors, which likely play a significant role in determining treatment adequacy.

## Conclusions

This study provides useful descriptive information about the adequacy of mental health care globally, using MAT as a “real-world” intermediate benchmark for treatment quality, which can be especially useful in low-income countries, where allocation of very limited resources must be prioritized for interventions that offer the greatest impact on treatment accessibility and effectiveness. Approximately two-thirds of treated cases met criteria for MAT. Treatment characteristics, particularly care provided by mental health specialists and across multiple sectors, were the strongest predictors of MAT. Additionally, patients’ perceived severity of their condition played a key role in engagement and treatment continuity, which should be considered when aiming to improve the quality of care delivered. To advance global mental health care, future research should focus on validating the MAT definition against clinical outcomes and further exploring provider and system-level factors that influence treatment adequacy.

## Supplementary Information

Additional file 1

## Data Availability

Access to the cross-national World Mental Health (WMH) data is governed by the organizations funding and responsible for survey data collection in each country. These organizations made data available to the WMH consortium through restricted data sharing agreements that do not allow us to release the data to third parties. The exception is that the U.S. data are available for secondary analysis via the Inter-University Consortium for Political and Social Research (ICPSR), http://www.icpsr.umich.edu/icpsrweb/ICPSR/series/00527.

## Abbreviations

APA: American psychological association
AOO: Age-of-onset
CAM: Complementary or alternative medicine
CIDI: Composite international diagnostic interview
DSM-IV: Diagnostic and statistical manual of mental disorders, fourth edition
NICE: National institute for health and care excellence
ICD-10: International classification of diseases 10th revision
IQR: Interquartile range
MAT: Minimally adequate treatment
RRs: Risk ratios
STROBE: Strengthening the reporting of observational studies in epidemiology
WMH: World mental health
WHO: World health organization
