# Supplementary material for: Predictors and barriers to minimally adequate treatment among treated individuals with mental disorders: results from the World Mental Health Surveys
Source: Int J Ment Health Syst. 2025 Dec 18;19:34. doi: 10.1186/s13033-025-00686-6 (PMC12720457; doi:10.1186/s13033-025-00686-6)
Supplement: Supplementary file 1 — Additional file 1 [file 13033_2025_686_MOESM1_ESM.docx]

**Additional File 1**

**Supplementary material for Pozuelo et al.,**

**“Predictors and barriers to minimally adequate treatment among treated individuals with mental disorders: Results from the World Mental Health Survey”**

| **Supplementary Table 1. World Mental Health sample characteristics by World Bank income categories*^a^*** | | | | | | | | |
| --- | --- | --- | --- | --- | --- | --- | --- | --- |
|  |  |  |  |  | **Sample size** | | |  |
| **Country by income category** | **Survey^b^** | **Sample characteristics^c^** | **Field dates** | **Age range** | **Part I** | **Part II** | **Part II and age ≤ 44^d^** | **Response rate^e^** |
| **I. Low- and middle-income countries** | | | | | | | | |
| Brazil - São Paulo | São Paulo Megacity | São Paulo metropolitan area. | 2005-8 | 18-93 | 5,037 | 2,942 | - | 81.3 |
| Bulgaria | NSHS | Nationally representative. | 2002-6 | 18-98 | 5,318 | 2,233 | 741 | 72.0 |
| Bulgaria – 2 | NSHS-2 | Nationally representative. | 2016-17 | 18-91 | 1,508 | 578 | - | 61.0 |
| Colombia | NSMH | All urban areas of the country (approximately 73% of the total national population). | 2003 | 18-65 | 4,426 | 2,381 | 1,731 | 87.7 |
| Colombia – Medellin | MMHHS | Medellin metropolitan area. | 2011-12 | 19-65 | 3,261 | 1,673 | - | 97.2 |
| Lebanon | LEBANON | Nationally representative. | 2002-3 | 18-94 | 2,857 | 1,031 | 595 | 70.0 |
| Mexico | M-NCS | All urban areas of the country (approximately 75% of the total national population). | 2001-2 | 18-65 | 5,782 | 2,362 | 1,736 | 76.6 |
| Nigeria | NSMHW | 21 of the 36 states in the country, representing 57% of the national population. The surveys were conducted in Yoruba, Igbo, Hausa and Efik languages. | 2002-4 | 18-100 | 6,752 | 2,143 | 1,203 | 79.3 |
| Peru | EMSMP | Five urban areas of the country (approximately 38% of the total national population). | 2004-5 | 18-65 | 3,930 | 1,801 | 1,287 | 90.2 |
| Romania | RMHS | Nationally representative. | 2005-6 | 18-96 | 2,357 | 2,357 | - | 70.9 |
| **TOTAL** |  |  |  |  | (41,228) | (19,501) | (7,293) | 79.8 |
| **II. High-income countries** | | | | | | | | |
| Argentina | AMHES | Eight largest urban areas of the country (approximately 50% of the total national population). | 2015 | 18-98 | 3,927 | 2,116 | - | 77.3 |
| Belgium | ESEMeD | Nationally representative. The sample was selected from a national register of Belgium residents. | 2001-2 | 18-95 | 2,419 | 1,043 | 486 | 50.6 |
| France | ESEMeD | Nationally representative. The sample was selected from a national list of households with listed telephone numbers. | 2001-2 | 18-97 | 2,894 | 1,436 | 727 | 45.9 |
| Germany | ESEMeD | Nationally representative. | 2002-3 | 19-95 | 3,555 | 1,323 | 621 | 57.8 |
| Israel | NHS | Nationally representative. | 2003-4 | 21-98 | 4,859 | 4,859 | - | 72.6 |
| Italy | ESMeD | Nationally representative. The sample was selected from municipality resident registries. | 2001-2 | 18-100 | 4,712 | 1,779 | 853 | 71.3 |
| Japan | WMHJ 2002-2006 | Eleven metropolitan areas. | 2002-6 | 20-98 | 4,129 | 1,682 | - | 55.1 |
| Netherlands | ESMeD | Nationally representative. The sample was selected from municipal postal registries. | 2002-3 | 18-95 | 2,372 | 1,094 | 516 | 56.4 |
| Northern Ireland | NISHS | Nationally representative. | 2005-8 | 18-97 | 4,340 | 1,986 | - | 68.4 |
| Poland | EZOP | Nationally representative. | 2010-11 | 18-65 | 10,081 | 4,000 | 2,276 | 50.4 |
| Poland – 2 | EZOP-2 | Nationally representative. | 2018-19 | 18-96 | 11,998 | 4,776 | - | 63.5 |
| Portugal | NMHS | Nationally representative. | 2008-9 | 18-81 | 3,849 | 2,060 | 1,070 | 57.3 |
| Spain | ESEMeD | Nationally representative. | 2001-2 | 18-98 | 5,473 | 2,121 | 960 | 78.6 |
| Spain-Murcia | PEGASUS-Murcia | Murcia region - regionally representative. | 2010-12 | 18-96 | 2,621 | 1,459 | - | 67.4 |
| United States | NCS-R | Nationally representative. | 2001-3 | 18-99 | 9,282 | 5,692 | 3,197 | 70.9 |
| **TOTAL** |  |  |  |  | (76,511) | (37,426) | (10,706) | 63.7 |
| **III. TOTAL** |  |  |  |  | (117,739) | (56,927) | (17,999) | 69.3 |
|  |  |  |  |  |  |  |  |  |

^a^Some of the WMH countries have moved into new income categories since the surveys were conducted. The income groupings above reflect the status of each country at the time of data collection. The current income category of each country is available at <http://data.worldbank.org/country> [1].

^b^NSHS (Bulgaria National Survey of Health and Stress); NSMH (The Colombian National Study of Mental Health); MMHHS (Medellín Mental Health Household Study); LEBANON (Lebanese Evaluation of the Burden of Ailments and Needs of the Nation); M-NCS (The Mexico National Comorbidity Survey); NSMHW (The Nigerian Survey of Mental Health and Wellbeing); EMSMP (La Encuesta Mundial de Salud Mental en el Peru); RMHS (Romania Mental Health Survey); AMHES (Argentina Mental Health Epidemiologic Survey); ESEMeD (The European Study Of The Epidemiology Of Mental Disorders); WMHJ2002-2006 (World Mental Health Japan Survey); NISHS (Northern Ireland Study of Health and Stress); EZOP (Epidemiology of Mental Disorders and Access to Care Survey); NMHS (Portugal National Mental Health Survey); PEGASUS-Murcia (Psychiatric Enquiry to General Population in Southeast Spain-Murcia); NCS-R (The US National Comorbidity Survey Replication).

^c^Most WMH surveys were based on stratified multistage clustered area probability household samples in which samples of areas equivalent to counties or municipalities in the US were selected in the first stage followed by one or more subsequent stages of geographic sampling (e.g., towns within counties, blocks within towns, households within blocks) to arrive at a sample of households, in each of which a listing of household members was created and one or two people were selected from this listing to be interviewed. No substitution was allowed when the originally sampled household resident could not be interviewed. These household samples were selected from Census area data in all countries other than France (where telephone directories were used to select households) and the Netherlands (where postal registries were used to select households). Several WMH surveys (Belgium, Germany, Italy, Poland, Poland 2, Spain-Murcia) used municipal, country resident or universal health-care registries to select respondents without listing households. The Japanese sample is the only totally un-clustered sample, with households randomly selected in each of the 11 metropolitan areas and one random respondent selected in each sample household. 16 of the 25 surveys are based on nationally representative household samples.

^d^Argentina, Brazil, Bulgaria 2, Colombia-Medellin, Israel, Japan, Northern Ireland, Poland 2, Romania, and Spain-Murcia did not have an age restricted Part 2 sample. All other surveys, with the exception of Nigeria (which was age restricted to ≤ 39) were age restricted to ≤ 44.

^e^The response rate is calculated as the ratio of the number of households in which an interview was completed to the number of households originally sampled, excluding from the denominator households known not to be eligible either because of being vacant at the time of initial contact or because the residents were unable to speak the designated languages of the survey. The weighted average response rate is 69.3%

| **Supplementary Table 2. Pooled within-country associations of socio-demographics with MAT among treated cases (n=3,538)^a^** | | | | | | | | |
| --- | --- | --- | --- | --- | --- | --- | --- | --- |
|  |  | |  |  | |  |  | |
|  | **Distribution** | |  | **Univariable** | |  | **Multivariable** | |
|  | **%** | **(SE)** |  | **RR** | **(95% CI)** |  | **RR** | **(95% CI)** |
| I. Sex |  |  |  |  |  |  |  |  |
| Female | 68.8 | (1.6) |  | 1.0 | (0.9-1.1) |  | 1.0 | (0.9-1.1) |
| Male | 31.2 | (1.6) |  | 1.0 | - |  | 1.0 | - |
| χ^2^_1_ |  | |  | 0.5 | |  | 0.2 | |
| II. Age |  |  |  |  |  |  |  |  |
| 18-29 | 21.1 | (1.4) |  | 1.0 | (0.9-1.2) |  | 1.1 | (0.9-1.3) |
| 30-44 | 37.6 | (1.2) |  | 1.0 | (0.9-1.2) |  | 1.1 | (0.9-1.2) |
| 45-59 | 31.8 | (1.3) |  | 1.0 | (0.9-1.2) |  | 1.0 | (0.9-1.2) |
| 60+ | 9.5 | (0.7) |  | 1.0 | - |  | 1.0 | - |
| χ^2^_3_ | - | |  | 0.3 | |  | 0.7 | |
| III. Education^b^ |  |  |  |  |  |  |  |  |
| Low | 16.9 | (1.0) |  | 1.0 | (0.8-1.1) |  | 0.9 | (0.8-1.1) |
| Low-average | 25.9 | (1.5) |  | 1.0 | (0.8-1.1) |  | 1.0 | (0.8-1.1) |
| High-average | 32.4 | (1.4) |  | 1.1 | (0.9-1.2) |  | 1.1 | (1.0-1.2) |
| High | 21.9 | (1.3) |  | 1.0 | - |  | 1.0 | - |
| χ^2^_3_ | - | |  | 3.6 | |  | 3.9 | |
| VI. Marital status |  |  |  |  |  |  |  |  |
| Married/cohabitating | 49.9 | (1.5) |  | 1.0 | (0.9-1.1) |  | 1.0 | (0.9-1.1) |
| Previously married | 24.3 | (1.2) |  | 1.1 | (1.0-1.3) |  | 1.2 | (1.0-1.4) |
| Never married | 25.9 | (1.4) |  | 1.0 | - |  | - | - |
| χ^2^_2_ | - | |  | 5.1 | |  | 5.7 | |
| V. Perceived need^c^ |  |  |  |  |  |  |  |  |
| Yes | 89.5 | (0.9) |  | 0.9 | (0.8-1.1) |  | 0.9 | (0.8-1.1) |
| No | 10.5 | (0.9) |  | 1.0 | - |  | 1.0 | - |
| χ^2^_1_ | - | |  | 0.7 | |  | 0.8 | |
|  |  | |  |  | |  |  | |

*Univariable* associations of each row predictor with minimally adequate treatment in a separate model controlling only for survey; *Multivariable* associations of all socio-demographic predictors with minimally adequate treatment in a single model controlling for survey; *%* the distribution of the socio-demographic predictors; *SE* the design-based standard error of % taking into consideration the weighting and geographic clustering of observations; *RR* relative risk of minimally adequate treatment as a function of the row predictor; *95% CI* the design-based 95% confidence interval of RR, taking into consideration the weighting and geographic clustering of observations.

^a^Pooled across all WMH surveys, with surveys weighted by sample size rather than by country population size.

^b^In quartiles defined by country-specific distributions (see [2] for details).

^c^Perceived need did not interact significantly with any of the socio-demographic predictors in the models and therefore was not used in any models except for the socio-demographic.

*Significant at the .05 level, two-sided design-based test.

| **Supplementary Table 3. Pooled within-country associations of lifetime treatment history with MAT among treated cases (n=3,538)^a^** | | | | | | | | | | | |
| --- | --- | --- | --- | --- | --- | --- | --- | --- | --- | --- | --- |
|  | **Distribution** | |  | **Univariable** | |  | **Multivariable** | |  | **Preliminary consolidated multivariable** | |
|  | **%** | **(SE)** |  | **RR** | **(95% CI)** |  | **RR** | **(95% CI)** |  | **RR** | **(95% CI)** |
| I. Lifetime Provider type |  |  |  |  |  |  |  |  |  |  |  |
| Psychiatrist | 55.9 | (1.4) |  | 1.2* | (1.1-1.3) |  | 1.1 | (0.9-1.5) |  | 0.9 | (0.8-1.1) |
| Other mental health | 52.0 | (1.6) |  | 1.3* | (1.2-1.5) |  | 1.3* | (1.0-1.7) |  | 1.1 | (0.9-1.2) |
| General medical | 52.0 | (1.4) |  | 0.9 | (0.8-1.0) |  | 0.9 | (0.7-1.2) |  | 1.0 | (0.9-1.1) |
| Human services | 14.8 | (1.1) |  | 1.0 | (0.9-1.1) |  | 0.9 | (0.7-1.2) |  | 1.0 | (0.9-1.1) |
| CAM | 18.5 | (1.1) |  | 0.9 | (0.8-1.1) |  | 0.9 | (0.6-1.2) |  | 0.9 | (0.8-1.0) |
| χ^2^_5_ | - | |  | 52.9* | |  | 34.2* | |  | 6.1 | |
| II. Number of past lifetime provider types | | |  |  |  |  |  |  |  |  |  |
| 1 | 22.1 | (1.3) |  | 1.0 | (0.9-1.2) |  | 1.0 | - |  | - | - |
| 2 | 22.3 | (1.2) |  | 1.3* | (1.1-1.4) |  | 1.0 | (0.8-1.4) |  | - | - |
| 3 | 23.3 | (1.6) |  | 1.2* | (1.1-1.5) |  | 1.0 | (0.6-1.7) |  | - | - |
| 4 | 9.0 | (0.8) |  | 1.4* | (1.2-1.6) |  | 1.2 | (0.5-2.6) |  | - | - |
| 5 | 4.1 | (0.7) |  | 1.4* | (1.2-1.8) |  | 1.4 | (0.5-3.8) |  | - | - |
| χ^2^_5/4_ | - | |  | 24.8* | |  | 6.8 | |  | - | |
| III. Lifetime treatment types |  |  |  |  |  |  |  |  |  |  |  |
| Medication-only | 11.6 | (1.1) |  | 1.1 | (0.9-1.3) |  | 1.0 | - |  | - | - |
| Psychotherapy-only^b^ | 15.5 | (1.1) |  | 1.0 | (0.9-1.2) |  | 1.1 | (0.9-1.3) |  | - | - |
| Combined | 53.7 | (1.4) |  | 1.3* | (1.1-1.5) |  | 1.2* | (1.0-1.4) |  | - | - |
| Neither | 19.2 | (1.0) |  | 1.0 | - |  | 1.0 | - |  | - | - |
| χ^2^_2_ | - | |  | 20.9* | |  | 4.7 | |  | - | |
| IV. Helpfulness of treatment |  |  |  |  |  |  |  |  |  |  |  |
| Helpful tx for any 12-mo dx | 62.8 | (1.3) |  | 1.2* | (1.1-1.4) |  | 0.8* | (0.7-1.0) |  | 1.1 | (0.9-1.2) |
| Unhelpful tx for any 12-mo dx | 13.0 | (1.0) |  | 1.2 | (1.0-1.4) |  | 0.8* | (0.7-1.0) |  | 1.0 | (0.9-1.2) |
| Only for other dx | 5.0 | (0.5) |  | 1.3* | (1.1-1.5) |  | 1.0 | - |  | 1.0 | - |
| No prior tx | 19.2 | (1.0) |  | 1.0 | - |  | 1.0 | - |  | 1.0 | - |
| χ^2^_2_ | - | |  | 8.3* | |  | 6.6* | |  | 1.5 | |
|  |  | |  |  | |  |  | |  |  | |

*Univariable* associations of each row predictor with minimally adequate treatment in a separate model controlling only for survey; *Multivariable* associations of all lifetime provider predictors with minimally adequate treatment in a single model controlling for survey; *Preliminary consolidated multivariable* associations of all prior treatment history predictors with minimally adequate treatment in a single model controlling for survey and disorder-related predictors and 12-month treatment history predictors; *RR* relative risk of minimally adequate treatment as a function of the row predictor; *95% CI* the design-based 95% confidence interval of RR, taking into consideration the weighting and geographic clustering of observations.

^a^Pooled across all WMH surveys, with surveys weighted by sample size rather than by country population size.

^b^Psychotherapy-only was created from the responses to this question: "Did you ever in your life have a session of psychological counseling or therapy lasting 30 minutes or longer with any type of professional?"  Those who said yes were asked their age and if it was > 2 years from their age at interview it was defined as "prior" psychotherapy.

*Significant at the .05 level, two-sided design-based test.

| **Supplementary Table 4. Barriers to receiving MAT among those who quit treatment** | | |
| --- | --- | --- |
|  | **Reasons for quitting treatment^a^** | |
| I. Types of barriers | **%** | **(SE)** |
| Low perceived severity | 69.5 | (2.7) |
| Financial | 22.3 | (2.0) |
| Other enabling barriers | 17.5 | (2.2) |
| Low perceived treatment effectiveness | 42.1 | (4.0) |
| Perceived stigma | 14.8 | (5.6) |
| II. Number of barriers |  |  |
| 0^b^ | 5.6 | (0.3) |
| 1 | 50.3 | (3.7) |
| 2 | 20.2 | (2.0) |
| 3 | 20.6 | (5.0) |
| 4 | 2.5 | (0.1) |
| 5 | 0.7 | (0.0) |
| (n) | (199) | |
|  |  | |

*Low perceived severity* reporting that a reason for not getting minimally adequate treatment due to quitting treatment before completion was either thinking that the problem would get better on its own, the problem not being very bothersome, a/o wanting to handle the problem on their own; *Financial* reporting that a reason for not getting minimally adequate treatment due to quitting treatment before completion was either that insurance would not pay for treatment a/o concerns about not being about to afford treatment; *Other enabling factors* reporting that a reason for not getting minimally adequate treatment due to quitting treatment before completion was either having problems with things like transportation or scheduling that made it hard to get to treatment, being unsure about where to go or who to see, thinking that treatment would take too much time or be inconvenient, a/o not being able to get an appointment; *Low perceived treatment effectiveness* reporting that a reason for not getting minimally adequate treatment due to quitting treatment before completion was either not being satisfied with available treatments, not thinking treatment will work, a/o having been in treatment in the past and not finding it helpful; *Perceived stigma* reporting that a reason for not getting minimally adequate treatment due to quitting treatment before completion was either concern about what people would think if they found out the patient was in treatment a/o worry about being involuntarily committed to a hospital; *%* proportion of respondents in the column total who reported the barrier or number of barriers in the row heading; *SE* design-based standard error of %.

^a^Pooled across all WMH surveys in the subsample of respondents with treatment contact who did not get minimally adequate treatment due to quitting their 12-month treatment before fruition and among those who were asked about reasons for quitting. This figure excluded the respondents who are classified as not receiving minimally adequate treatment due to reported inadequate treatment (n=180) or since they are still in treatment with less than 8 visits (n=814).

^b^Respondents with 0 reasons responded “No” to all reasons (n=13 of those who quit treatment), those who were missing on all reasons were dropping from the population of individuals who quit (n=32 of those who quit treatment).

**References**

1. World Bank. World Bank Open Data [Internet]. 2024 [cited 2024 Apr 10]. Available from: <https://data.worldbank.org/>
2. Evans-Lacko S, Aguilar-Gaxiola S, Al-Hamzawi A, Alonso J, Benjet C, Bruffaerts R et al. Socio-economic variations in the mental health treatment gap for people with anxiety, mood, and substance use disorders: Results from the WHO World Mental Health (WMH) surveys. Psychol Med. 2018;48(9):1560-71. <http://doi.org/10.1017/s0033291717003336>.
